# Genetic diversity and population structure assessed by SSR and SNP markers in a large germplasm collection of grape

**DOI:** 10.1186/1471-2229-13-39

**Published:** 2013-03-07

**Authors:** Francesco Emanuelli, Silvia Lorenzi, Lukasz Grzeskowiak, Valentina Catalano, Marco Stefanini, Michela Troggio, Sean Myles, José M Martinez-Zapater, Eva Zyprian, Flavia M Moreira, M Stella Grando

**Affiliations:** 1 Department of Genomics and Biology of Fruit Crops, IASMA Research and Innovation Centre, Fondazione Edmund Mach - Via E. Mach 1, San Michele all'Adige, TN, 38010, Italy; 2 Department of Plant and Animal Sciences, Faculty of Agriculture, Dalhousie University, Truro, Nova Scotia, B2N 5E3, Canada; 3 Instituto de Ciencias de la Vid y del Vino (CSIC, UR, Gobierno de La Rioja), C/ Madre de dios 51, Logroño, 26006, Spain; 4 JKI Institute for Grapevine Breeding Geilweilerhof, Siebeldingen, 76833, Germany; 5 Instituto Federal de Santa Catarina, Rua José Lino Kretzer 608 - Praia Comprida, São José, Santa Catarina, 88130-310, Brasil

**Keywords:** Grapevine, Diversity pattern, Population structure, Phenotypic variation, Core collections, *Vitis* spp

## Abstract

**Background:**

The economic importance of grapevine has driven significant efforts in genomics to accelerate the exploitation of *Vitis* resources for development of new cultivars. However, although a large number of clonally propagated accessions are maintained in grape germplasm collections worldwide, their use for crop improvement is limited by the scarcity of information on genetic diversity, population structure and proper phenotypic assessment. The identification of representative and manageable subset of accessions would facilitate access to the diversity available in large collections. A genome-wide germplasm characterization using molecular markers can offer reliable tools for adjusting the quality and representativeness of such core samples.

**Results:**

We investigated patterns of molecular diversity at 22 common microsatellite loci and 384 single nucleotide polymorphisms (SNPs) in 2273 accessions of domesticated grapevine *V. vinifera* ssp. *sativa*, its wild relative *V. vinifera* ssp. *sylvestris*, interspecific hybrid cultivars and rootstocks. Despite the large number of putative duplicates and extensive clonal relationships among the accessions, we observed high level of genetic variation. In the total germplasm collection the average genetic diversity, as quantified by the expected heterozygosity, was higher for SSR loci (0.81) than for SNPs (0.34). The analysis of the genetic structure in the grape germplasm collection revealed several levels of stratification. The primary division was between accessions of *V. vinifera* and non-*vinifera*, followed by the distinction between wild and domesticated grapevine. Intra-specific subgroups were detected within cultivated grapevine representing different eco-geographic groups. The comparison of a phenological core collection and genetic core collections showed that the latter retained more genetic diversity, while maintaining a similar phenotypic variability.

**Conclusions:**

The comprehensive molecular characterization of our grape germplasm collection contributes to the knowledge about levels and distribution of genetic diversity in the existing resources of *Vitis* and provides insights into genetic subdivision within the European germplasm. Genotypic and phenotypic information compared in this study may efficiently guide further exploration of this diversity for facilitating its practical use.

## Background

The genus *Vitis* contains about 60 species, or more strictly ecospecies, since there are no genetic barriers within the genus and the species are inter-fertile. They have a primarily temperate zone distribution, occurring extensively in the Northern Hemisphere. The leading cultivated species by far is *V. vinifera* L. ssp. *sativa (*or *vinifera)*, and its wild form *V. vinifera* L. ssp. *sylvestris* represents the only *Vitis* taxon naturally found in Europe. In contrast, numerous species of this genus are indigenous to North America and East Asia. Although these wild species are only peripherally used for human consumption, they are of great economic importance as a source for resistance breeding and as rootstocks for the highly susceptible *V. vinifera*. Since the beginnings of cultivation, desirable forms of the wild grapevine and spontaneous mutants within cultivated populations have been selected and preserved by vegetative propagation. Additional cultivars have been developed by both deliberate and spontaneous interspecific as well as intraspecific breeding [1]. A significant reduction of genetic diversity in both cultivated and wild grapevines occurred when the phylloxera insect was brought to Europe from America about 150 years ago. European vineyards were saved from extinction by the introduction of several native American, non-*vinifera Vitis* species, which were used as rootstocks and for breeding disease resistant interspecific hybrids [2]. Over the past few decades the cultivated grapevine has experienced another drastic reduction of diversity, resulting in the disappearance of old local varieties, and the increased focus of global wine companies on only a few major cultivars. Likewise, genetic variation of the wild *V. vinifera* species has diminished due to loss of natural habitat. On the other hand, in the recent past, many conservation programs of genetic resources have been conducted in grapevine growing countries. As a result, a significant number of minor varieties have been collected and preserved in field collections. However, due to the long time required for field experiments and the lack of information on genetic variation, research efforts that would facilitate the use of existing collections for crop improvement have not been as frequent as the conservation activities.

Molecular characterization is now the favored way to quantify variation within germplasm samples [3-5]. For instance, microsatellites (simple sequence repeats, SSR), because of their polymorphism, reproducibility, and codominant nature, have become the markers of choice for compilation, standardization and exchange of information concerning grapevine genetic resources [6]. Recently SSRs were applied in several surveys of entire germplasm collections [7-9]. These studies provided a broader estimation of genetic diversity in each collection and found a high degree of clonal relationships, synonyms, homonyms, and curation errors. Similar conclusions on the naming accuracy were achieved in the analysis of the USDA grape germplasm collection, using a genome-wide SNP genotyping approach [10]. The authors evaluated haplotype diversity, pattern of population structure and the decay of linkage disequilibrium in *V. vinifera* accessions, with a set of 5,387 SNPs. Results of the survey suggest that although substantial genetic diversity has been maintained in the grape following domestication, there has been a limited exploration of this diversity. Since it is still unclear to what extent these collections represent an unbiased sample of the worldwide genetic variation, further broad studies of grapevine germplasm are required, as well as the development of a manageable set of materials that will facilitate access to this variation. The present study is part of an effort to characterize and to dissect the genetic structure of one of the largest collections of grape germplasm in Europe, which maintains, amongst the 2700 accessions, hundreds of putative wild *V. vinifera* individuals and selections of post-phylloxera breeding materials. Our aim is to maximize the potential contribution of the collection dataset to the development of an international database and the creation of composite core collections. We applied the SSR descriptors chosen for the European *Vitis* Database [11], the SSRs employed to genotype the largest grape repository in the world [9], and 384 SNPs spread throughout the genome, which included the set of markers proposed for grapevine cultivar identification [12]. This allowed us 1) to examine the level of genetic diversity, structure and differentiation within the germplasm collection, comparing the usefulness of different marker systems; 2) to sort out genetic core collections from the dataset of *V. vinifera* cultivars and contrast their genetic variation with that of a sample representative of the collection’s phenological variation, with the intention of justifying a contribution of these samples to association studies.

## Results and discussion

### Genetic characterization of a *Vitis* germplasm collection

A set of ten microsatellites combined in four multiplex panels, including the standard set of markers for genetic identification, was used in a first step to analyse 2273 accessions and to compare their genotypes. Accessions were classified in four different categories: Sativa (*V. vinifera* ssp. *sativa*), Sylvestris (*V. vinifera* ssp. *sylvestris)*, Hybrids (interspecific hybrids of *Vitis* used for fruit production) and Rootstocks (rootstock varieties including wild non-*vinifera Vitis* species). Similar sets of markers proved a high discriminating capacity for grapevine varieties [7,9], and this was supported in the present study by a low cumulative probability of identity (PI) for the ten SSR loci: 10^-15^. A total of 713 multilocus SSR genotypes were represented by only one accession in the whole collection (Table 1). The other 1560 accessions possessed non-unique profile of microsatellite markers and were represented by 372 different genotypes, bringing the number of distinct SSR profiles to 1085. Approximately half of the collection (52%, 1188 accessions) was composed of redundant germplasm. The largest number of putative duplicates was observed within Sativa and Rootstocks, with many examples of different names being used for the same variety or clonal variants. In most cases the redundant genotypes were in agreement with expectations, since they corresponded either to synonymous cultivars, sports (spontaneous somatic mutants) or clonal selections which are not likely to be differentiated from their original cultivar using a few molecular markers. Examples are provided for 63 groups of accessions with identical SSR profiles that included true-to-type Italian varieties (Additional file 1: Table S1). In viticulture, grapevine varieties are considered to consist of clones that share common morphological traits. When clones of the same variety have phenotypes different enough to be grown for the production of different wines, they are grouped into different cultivars [13]. Thanks to their high genetic similarity level, clones with differing phenotypic characters could provide material suitable for functional genomic studies. For these reasons accessions sharing the same SSR profile are worthy of further morphological evaluation before being considered for elimination from the collection. However, sometimes the cultivar names associated with each DNA sample were clearly incorrect (Additional file 1: Table S1). To determine the causes of naming inaccuracies, analyses were repeated using the same DNA extraction as well as independent DNA extractions for each plant combined with visual inspections in the field. We concluded that the cases of cultivar misidentification are likely often due to curation errors, which are common in germplasm collections, e.g. introduction of similar material under different names from different donors.

**Table 1:** Level of redundancy and number of multilocus genotypes identified using 10 SSRs within the entire FEM germplasm collection and its four grape subpopulations

**Population**	**Accessions analyzed**	**Different SSR genotypes**	**SSR genotypes represented by one accession**	**SSR genotypes represented by two or more accessions**	**Average number of accessions with identical SSR genotype**
Sativa	1659	733	450	283	4.3
Sylvestris	177	139	120	19	3.0
Hybrids	127	86	65	21	3.0
Rootstocks	310	127	78	49	4.7
Total	2273	1085	713	372	4.2

### Genetic diversity

The set of 1085 distinct genotypes identified with ten SSR markers was further characterized using 12 additional SSRs and 384 genome-wide SNPs. The analysis of polymorphism in this sample set showed that both the microsatellites and SNP markers were informative. All the 22 SSR loci were very polymorphic among grapevine accessions, with a large number of alleles detected. In contrast, from the 384 SNP loci initially chosen, 31 were discarded because many values were missing while 353 were proven to be polymorphic and showed the presence of low and intermediate frequency alleles. For instance, in the whole collection, the number of different alleles (A) for the SSRs was 499 and ranged from 9 to 42 per locus, with an average of 22.68. The allele frequency at the SSR loci was either low or high, and this can explain the moderate effective number of alleles, which measures evenness of the most common alleles at the tested loci. It varied between 2.12 and 10.11, with an average of 6.19. For SNPs the average number of effective alleles was 1.58 and 83% of the 353 variable loci showed minor allele frequency (MAF) values > 0.1. The observed and expected heterozygosities, based on SSR markers, were 0.74 and 0.81, respectively, and these were more than twice higher than the values calculated for SNPs (0.30 and 0.34, respectively). The overall fixation index was similar for both marker systems (0.09). These parameters are summarized in Table 2 and in Additional file 1: Table S5.

**Table 2:** Summary statistics of genetic variation at 22 SSR loci and 353 SNP loci in the entire FEM germplasm collection and its four grape subpopulations

**Markers**	**Sample**	**N**	**n**	**A**	**a**	**A**_**E**_	**H**_**E**_	**H**_**O**_	**F**	**MAF**
SSR	Total	1085	1036.7	499	22.682	6.191	0.814	0.743	0.090	-
	Sativa	733	715.5	362	16.455	5.292	0.78	0.761	0.025	-
	Sylvestris	139	136.5	234	10.636	3.618	0.699	0.627	0.104	-
	Rootstocks	127	110.3	412	18.727	8.199	0.838	0.734	0.124	-
	Hybrids	86	74.5	294	13.364	5.682	0.81	0.796	0.011	-
SNP	Total	1072	1027.0	706	2.000	1.588	0.344	0.309	0.093	0.25846
	Sativa	728	703.1	704	1.994	1.589	0.345	0.349	−0.005	0.25809
	Sylvestris	137	131.8	687	1.946	1.421	0.266	0.251	0.046	0.19964
	Rootstocks	122	111.0	669	1.895	1.157	0.116	0.099	0.090	0.08193
	Hybrids	85	81.0	696	1.972	1.565	0.335	0.337	−0.014	0.25668

Total – entire germplasm collection (pooled sample treated as a single population); N – sample size; n – mean sample size over loci; A – number of different alleles; a – mean number of alleles per locus; A_E_ – effective number of alleles; H_E_ – unbiased expected heterozygosity; H_O_ – observed heterozygosity; F – fixation index (inbreeding coefficient); MAF – minor allele frequency.

When considering the four collection subsets (Sativa, Sylvestris, Rootstocks and Hybrids), the diversity parameters were different, compared to those estimated for the total collection. The number of alleles ranged from 234 in Sylvestris to 412 in Rootstocks for SSR loci, and from 669 in Rootstocks to 704 in Sativa for SNP loci. The average MAF of SNPs in Sativa and Hybrids was similar to that calculated for the total collection (0.25), while in Rootstocks and Sylvestris this value was only 0.08 and 0.19, respectively. The average effective number of alleles for SSR loci ranged from 3.61 in Sylvestris to 8.19 in Rootstocks, while these numbers for SNP loci were from 1.15 in Rootstocks to 1.58 in Sativa. The expected heterozygosity estimates of the subsets ranged from 0.69 (Sylvestris) to 0.83 (Rootstocks) for the SSRs and from 0.11 (Rootstocks) to 0.34 (Sativa) for the SNPs. The fixation index F ranged from 0.01 and −0.01 in Hybrids to 0.12 and 0.09 in Rootstocks, for the SSRs and SNPs, respectively. The SSR markers have been used to characterize diversity among cultivars from most of the regions of grape cultivation. The data from those studies are difficult to compare, because the number of accessions and the marker loci used are very different. However, similar to our survey, the other studies showed that the SSR diversity within *V. vinifera* is very high. In different analyses, the number of alleles per locus in Sativa varied from 8 to 11 for sample sizes ranging from 58 to 366 individuals and H_E_ ranged from 0.62 to 0.85 with an average of 0.76 [7,8,14-17]. For instance, Laucou et al. [9], using 20 SSRs on 2323 cultivated *V. vinifera* accessions (Sativa), revealed an average number of 16.9 alleles per locus (6–36) and the expected heterozygosity of 0.76. Likewise, although that analysis was intended for cultivated grapevines, the authors included wild grape accessions (Sylvestris), as well as accessions resulted from interspecific crosses with North American *Vitis* spp. (Hybrids and Rootstocks). The subset of Rootstocks in those studies also revealed the highest number of alleles (405) and the highest heterozygosity (0.86), in spite of their relatively small sample size compared to the subset of Sativa. The Sylvestris sample in this study [9] presented the lowest number of alleles (203) and heterozygosity (0.62). We have observed a similar trend in our survey: the set of Rootstocks displayed the highest number of different SSR alleles (412). Sativa was less diverse than the set of Rootstocks and more diverse than Sylvestris. Fixation index was low in Sativa and Hybrids collections (0.02 and 0.01, respectively), compared to Sylvestris and Rootstocks subsets (0.10 and 0.12, respectively). Diversity in Sylvestris was lower than in the cultivated grape, because of the small number of unique individuals available in the collection. This supports a previous hypothesis that suggested the scarcity of this endangered subspecies with small populations results in higher inbreeding rates [1]. The lower number of SSR alleles in Hybrids was likely due to a low sample size, however the level of heterozygosity (0.8) is consistent with former observations [9,18].

Previous studies on SNP variation in grapevine concerned mainly cultivated grapevines and reported a similar level of diversity. In the survey of 1573 SNPs from a group of 11 grape genotypes corresponding to nine ancient unrelated cultivars and two wild grapevines, the expected heterozygosity (H_E_) ranged from 0 to 0.66 with a mean value of 0.30 [19]. Likewise, in a set of 48 SNPs from a sample containing 151 non-redundant cultivars, H_E_ was 0.404 [12].

It was shown that level of diversity quantified by heterozygosity based on SNPs is around two times lower than that estimated for SSR markers [20,21]. This potential disadvantage of SNP can be overcome either by using a large number of markers or by considering haplotypes structure for each locus instead of single SNPs [10,19]. The differences between SNPs and SSRs in levels of genetic diversity result from mutational properties of these two marker types. Because of the nature of SNP markers, we observed a smaller proportion of rare alleles in the frequency distribution of the SNP data compared to the SSR data. The intermediate frequency alleles in the SNP loci could also be the consequence of ascertainment bias, which is the bias introduced when loci are identified in a small panel of accessions that do not represent the full genetic variation of a genus or species [22,23]. Moreover, the current high throughput genotyping of SNPs is based on measuring the relative signal strength of two expected alleles. However, in a population different allele types may exist at any locus and this unknown or “null” allele can interfere with exact genotyping of the expected alleles [24]. Since most of the SNPs used in this study were discovered in *V. vinifera*, this may explain the lower level of diversity in our dataset Rootstocks. Here the expected heterozygosity based on SNP genotypes was around seven times lower, compared to that from microsatellite loci (0.11 and 0.83 respectively). In contrast, several published studies indicate good transferability of SSR markers amongst *Vitis* species [25-29]. However, the SSR diversity may be an underestimation since sequencing of some microsatellite loci suggested that the polymorphism did not correspond only to a variation in the number of repeats, but also to changes in their architecture and the flanking regions with substitutions and long indels [29-34].

### Construction of a genetic core collection of *V. vinifera* sativa

The purpose of developing genetic core collections is to provide a restricted set of accessions, feasible to handle, and representing the genetic variability among individuals in a large source of germplasm. Genetic core collections were constructed to maximize the allelic diversity among Sativa accessions based on microsatellites, as these markers have been shown to provide greater information content compared to SNP markers. Based on the M-method, fifty eight cultivars (core G-58) were sufficient to capture all the 274 alleles occurring in more than 0.5% of the samples analyzed. The core G-58 was then used to design the final genetic core collection retaining 100% of SSR diversity, i.e. 362 alleles. The optimal size of this core was 110 individuals (core G-110), thus 52 accessions were added at this step to retain 88 rare alleles. The M-method sampling strategy showed a superior efficiency compared to random sampling. In particular core G-58 and core G-110 retained 45 (274 vs 229) and 101 (362 vs 261) more alleles compared to random cores of the same size (Additional file 2). These results show that only a small number of accessions are needed to retain the most frequent alleles as well as the whole allelic diversity (8% and 15% of cultivated grapevines in G-58 and G-110, respectively). A previous genetic core collection developed for cultivated grapevine by Le Cunff et al. [35] using the M-strategy required fewer individuals (92, i.e. 4%) to capture the total allelic diversity of the whole collection (326 alleles). We can assume that the high level of heterozygosity in grapevine is the major factor leading to capture all the genetic diversity with such a small number of individuals. Indeed, similar experiments have required 18% and 31% of individuals to retain the whole genetic diversity for *A. thaliana* and *M. truncatula,* respectively [36,37].

### Diversity of genetic and phenological core collections

Molecular marker diversity retained in the genetic core collections were compared with those of the whole Sativa germplasm collection and of the subset of 163 cultivars (core P) which represents the phenological variation of Sativa accessions with regard to time of budburst, flowering, *véraison* and full ripening (as described in Methods). Despite its size, the core P was shown to retain 2% less SSR diversity than the core G-58, and thus lacked a quarter of the alleles found in the whole Sativa germplasm (Table 3). On the other hand, when the 704 SNP alleles detected in the Sativa collection were considered, all three core collections have been shown to contain almost the whole diversity, with the core P and the core G-58 lacking only 2 and 3 alleles, respectively. Random sampling of 58 accessions (mean of ten replicates) resulted in retaining only 63% of the total SSR diversity, but retained 701 out of 704 SNP alleles. Likewise, Hamblin et al. [38] found in different small core sets of maize accessions a much higher percent of SNP alleles captured, compared to SSR alleles, as a consequence of their lower allelic richness.

**Table 3:** Descriptive statistics of SSR and SNP diversity within phenological (Core P) and genetic (Core G-58, Core G-110) core collections with the percentage of alleles retained from the entire germplasm collection of cultivated grapevine (Sativa)

**Sample**	**N**	**SSR**				**SNP**			
		**A**	**Alleles retained**	**H**_**E**_	**H**_**O**_	**A**	**Alleles retained**	**H**_**E**_	**H**_**O**_
Sativa	733#	362	100%	0.78	0.761	704	100%	0.345	0.349
Core P	163	267	74%	0.773	0.758	702	99.7%	0.345	0.349
Core G-58	58	274*	76%	0.813	0.773	701	99.6%	0.344	0.346
Core G-110	110	362	100%	0.815	0.774	704	100%	0.344	0.341
Random core G-58R §	58	229	63%	0.779	0.764	701	99.6%	0.347	0.352

N – sample size; A – number of different alleles; H_E_ – unbiased expected heterozygosity; H_O_ – observed heterozygosity; * minor allele frequency > 0.5%; # sample size for the SNP analysis was 728 individuals; § average of 10 randomizations.

**Figure 1:**
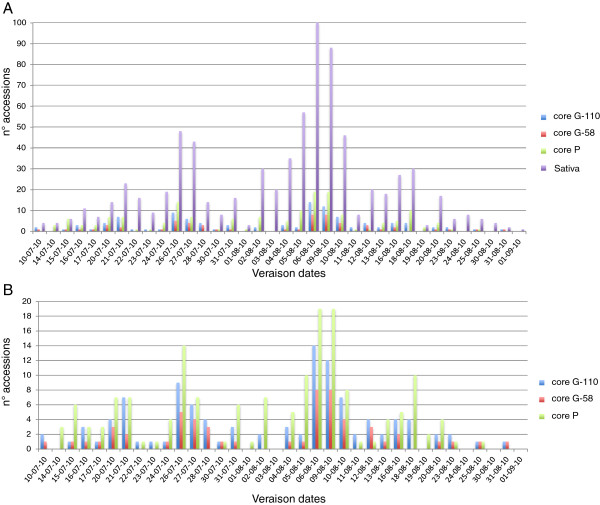
**Distribution of the ripening onset (*véraison*) of *V. vinifera *ssp. *sativa.*** Results of monitoring of 733 individuals from the FEM grape germplasm collection in 2010. Each bar represents the number of cultivars with fruits starting to soften and changing color in a given day/month. (**A**) and (**B**): comparison of all cultivated grapevine accessions (Sativa) with genetic (core G-58, core G-110) and phenological (core P) collections.

To explore the phenotypic diversity available in the genetic core collections and in the core P, the onset of ripening was recorded during summer in 2010 for all 733 Sativa accessions. This developmental stage, known in viticulture as “*véraison*”, represents the transition from berry growth to berry ripening, when berries start to soften and to change colour. Based on the date of the onset of ripening the Sativa accessions were grouped into 36 *véraison* classes which span almost two months, underlying a high phenotypic diversity (Figure 1). A similar distribution of the trait is visible for all sample sets, with a high proportion (81%) of the total *véraison* variability retained both in the core P and in the core G-110, and 64% retained in the core G-58. Despite the fact that core P included two more intermediate classes compared to core G-110 and seven more compared to core G-58, both G-core collections outperformed the core P in terms of extreme phenotypic classes. Four phenotypic classes identified in the whole Sativa collection were not retained in the G-core nor in the core P collections, but phenology shifts may be expected due to year-to-year variation. Altogether the results underline the capacity of both genetic cores to represent phenotypic variability at least for a key trait in the annual cycle of the vine, suggesting a potential contribution of the core germplasm to panels formed for genetic association studies, as shown for grape by Emanuelli et al. [39] and Fournier Level et al. [40].

### Population structure

**Figure 2:**
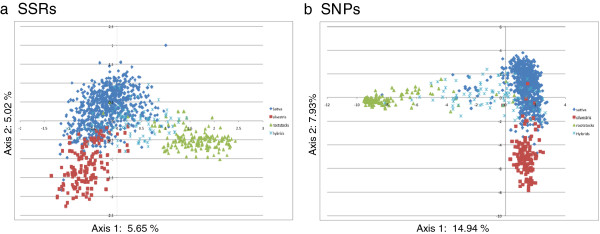
**Scatter plot from a PCA.** Principal component analysis of the FEM grape germplasm collection based on (**a**) 22 SSR loci and (**b**) 353 SNP loci.

The genetic structure of the whole germplasm collection was analyzed using PCA and STRUCTURE. The PCA approach based on allele frequencies of the SSR or SNP markers showed in both cases a clear differentiation between the two grapevine subspecies and the interspecific hybrid accessions, despite the presence of some overlapping zones (Figure 2). The first axes explained 5.65% and 14.94% of the overall variance for SSRs and SNPs, respectively, and separated *V. vinifera* genotypes from the Rootstocks. Within *vinifera*, the distinction between Sativa and Sylvestris was displayed on the second axes (5.02% for SSRs and 7.93% for SNPs), although a clear overlapping zone between wild and cultivated genotypes can be seen. A similar result was found by Laucou et al. [9] with Sylvestris germplasm originating from Western and Central Europe or the Maghreb (Northwest Africa), while almost all wild grapevine samples analyzed in the present study were collected from the Italian Peninsula. These findings would provide support for the occurrence of gene flow between wild and cultivated grapevine as reported previously by de Andrés et al. [41], although it cannot be excluded that a certain degree of similarity is common between the two subspecies. The same genetic divergence among samples was observed using the Principal Coordinate Analysis (PCoA) approach based on a genetic distance matrix with data standardization, where the first axes explained 38.51% and 53.10% of variance and the second axes 21.29% and 23.56%, for SSR and SNP marker loci respectively (data not shown).

Linkage disequilibrium between SNP loci was low (r^2^ < 0.2), which is consistent with previous findings [10,19]. Such level of LD is unlikely to affect the analysis of population structure. Both SSR and SNP datasets were independently used for the model-based Bayesian clustering method as implemented in STRUCTURE. The most likely number of clusters (K) was evaluated considering the plateau criterion proposed by Pritchard et al. [42], using the non-parametric Wilcoxon test [43] and the ΔK method [44] (Additional file 3 and Additional file 4). For the SSR dataset the mean log-likelihood curve attained a maximum value around K = 6, beyond which the mean log-likelihood values reached a plateau and the standard deviations associated with the estimates increased. In contrast, for the SNP dataset the mean log-likelihood curve did not reach a plateau and the standard deviations did not increase drastically. The aspect of consistency among different simulations within each preset K can also be visualized through the similarity coefficient between different runs for each preset K according to Nordborg et al. [45]. For both datasets the mean similarity coefficients among different simulations decreased for K larger than 5. The Wilcoxon test determined that best K is 6 for the SSR dataset and 5 for the SNP dataset. This test was also significant at K > 7 for the SNP data, reflecting a continuous increase of the likelihood values with respect to K. When more than five inferred populations are considered, no individual was strongly assigned (Q > 0.8 for SSRs or Q > 0.65 for SNPs) to the additional inferred populations. The ΔK criterion suggested by Evanno et al. [44] gave the highest value at two groups both for SSR and SNP loci, although peaks of ΔK were found also at K = 3 (for both SSRs and SNPs) and K = 6 (for SSRs only). This method is known to give rise to the first structural level in the data [46] and in the present study has led to discriminate the *Vitis* non-*vinifera* accessions (used as rootstocks) from the *V. vinifera* accessions (Sativa and Sylvestris), in agreement with the results displayed by PCA (Figure 2).

Hybrids are interspecific selections developed by crossing wild American species resistant to phylloxera, downy mildew or powdery mildew with European grapevine varieties. Several backcrosses with *V. vinifera* cultivars were required, especially for direct producer hybrids (ungrafted) to obtain superior wine grape cultivars. Accordingly, in the present study, Hybrids showed mixed ancestry with high admixture proportions of Sativa, ranging from 0.6 to 0.8.

**Figure 3:**
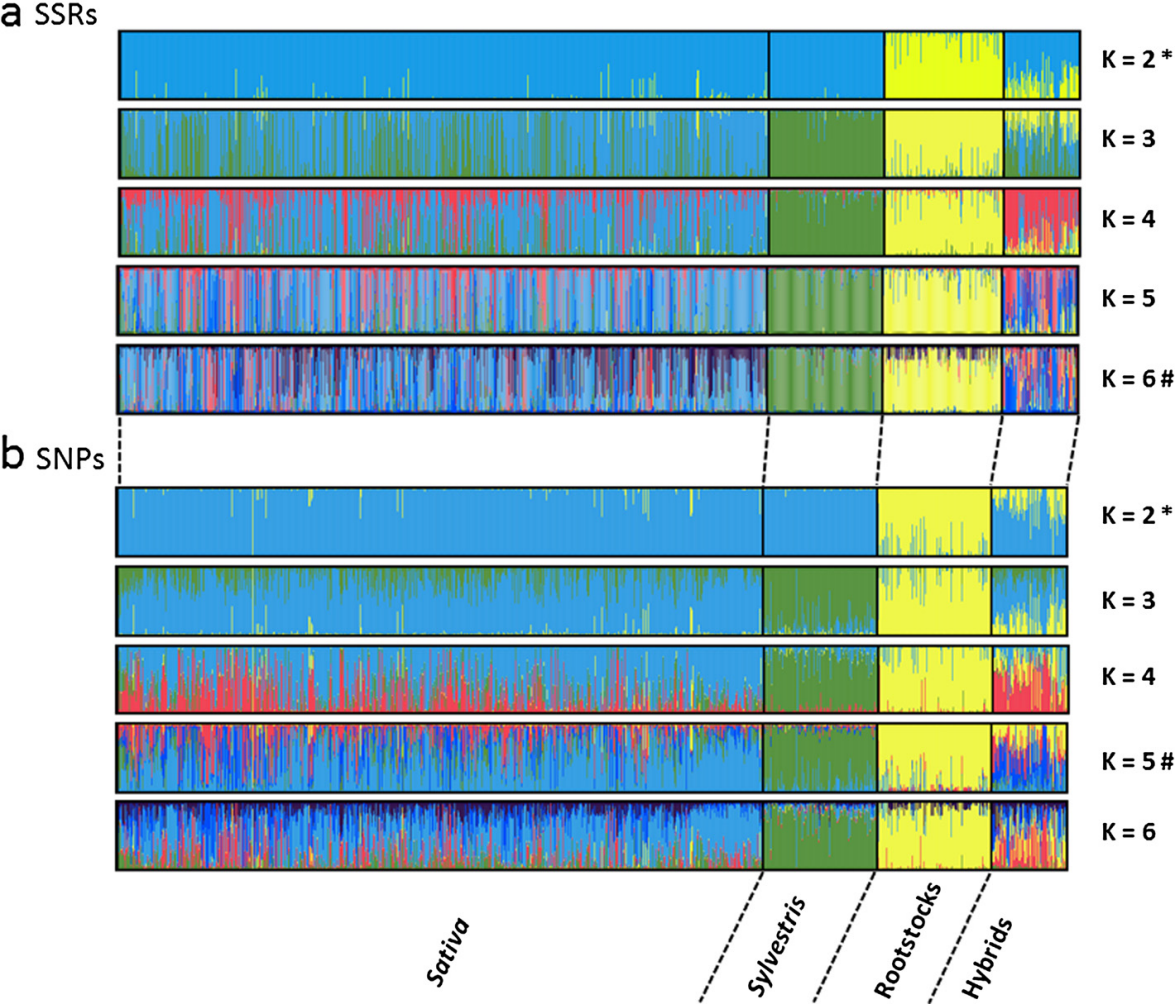
**Inferred population structure of the collection using the model-based program STRUCTURE **[47]**.** Plots generated with the DISTRUCT software based on the Q-matrix consensus permuted across 10 replications for K = 2 to K = 6 using the CLUMPP software. Each accession’s genome is represented by a single vertical line, which is partitioned into coloured segments in proportion to the estimated membership in the two to six subpopulations. On the y-axes is the likelihood of assignment to any given cluster K. Black line separates individuals of four predefined groups (see Table 1). (**a**) 22 SSR loci, 1085 individuals, (**b**) 353 SNP loci, 1072 individuals. * best K choice based on the ΔK method [44]. # best K choice based on the non-parametric Wilcoxon test.

Since different K values were detected with different methods using both marker data sets, the inferred population structure of the *Vitis* collection is shown for K ranging from 2 to 6 (Figure 3). At K = 2 the cultivated subspecies Sativa is not separated from its putative wild progenitor, while at K = 3 wild accessions are clearly clustered as a distinct subpopulation for both marker datasets. Using the SSR information some Sativa accessions remained grouped with Sylvestris samples at K =3, with ancestry up to 0.980. However at higher K values these accessions were re-sorted into a distinct cluster within Sativa. The result underlines a closer relationship of some cultivated accessions with the wild samples stressing again the possible occurrence of hybridization. From K = 4 to K = 6 the STRUCTURE software detected subpopulations only within Sativa and Hybrids clusters. At K = 5 Sativa accessions were divided into three groups: S1 (Mediterranean wine and table grapes), S2 (muscat-flavored wine and table grapes) and S3 (wine grapes from Central Europe). Similar clustering results were detected in core G-58 and core G-110, with group S1 being the most represented (31 and 52 accessions, respectively) followed by groups S2 (6 and 10 accessions) and S3 (5 and 8 accessions). This pointed out a clear genetic stratification also within the core collections that should be taken into account when designing genetic association studies.

Both SNPs and SSRs performed well in detecting the main subpopulations (K = 3): *V. vinifera* (Sativa and Sylvestris) and Rootstocks (*Vitis* ssp.). Except for K = 2, where both marker types showed a high percentage of individuals assigned to populations, all assignment percentages for the SNP dataset were lower than for the SSR dataset (Additional file 1: Table S6).

Differences in assignment percentages between SSR and SNP markers were also reported by independent studies in various maize germplasms [38,48,49] and were attributed to the greater information content of SSRs [50]. According to Laval et al. [51] (*k-*1) times more biallelic markers should theoretically achieve the same genetic distance accuracy as an SSR set of *k* alleles. In our study an average of about 22 alleles per SSR marker was found, thus [(22–1)*22] = 462 SNP markers should be needed to resolve all the relationships that have been detected using SSR markers; that would mean 109 SNPs in addition to the 353 finally used. Increasing the number of SNP markers will probably improve the inference of population structure, even outperforming the results obtained with SSRs, since using the SNP dataset standard deviations of L(K) were smaller even at high values of K. Nevertheless, the models of either 2 main groups (ΔK method) or 5–6 subpopulations (Wilcoxon test) could be supported by both marker datasets while the distinction between Sativa and Sylvestris was better resolved by using the SNP markers. To understand how comparable are the structure outputs based on SSRs and SNPs, the level of membership correlation was investigated assuming five populations. This was because both inter-specific (S5 and S4, Sylvestris and Rootstocks respectively) and intra-specific (Sativa: S1, S2, S3) subdivisions were detected at K = 5 and, as stated above, no individual was strongly assigned to the additional inferred populations for K > 5. The relationship between membership in the S1, S2, S3, S4 and S5 populations based on SSRs and membership based on SNPs were plotted. Correlations were strong for S5 and S4 (R^2^ = 0.93 and 0.86, respectively) and moderate for S1, S2, S3 (R^2^ = 0.74, 0.63 and 0.57 respectively) but there was clearly much more spread along the x-axes (SNPs) than along the y-axes (SSRs). Finally, the classification in five groups was in agreement with NJ analysis since individuals assigned to the same genetic group tended to be close together in the NJ trees (Additional file 5).

### Hierarchical population structure

**Figure 4:**
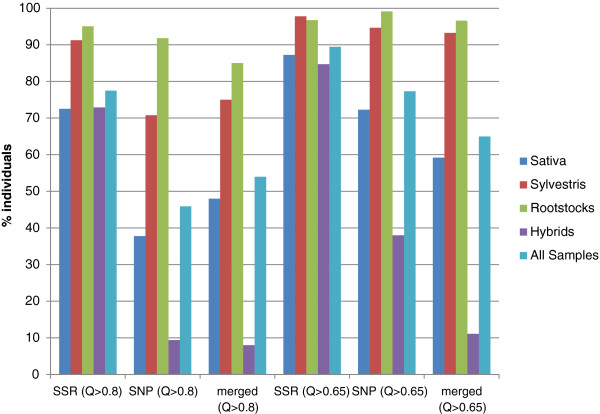
**Comparison of membership in germplasm clusters based on marker class.** The percent of individual assigned at K = 5 with different thresholds of membership coefficient (Q > 0.8 and Q > 0.65) is reported for each marker class separately and for the common accessions assigned using both marker datasets (merged).

**Figure 5:**
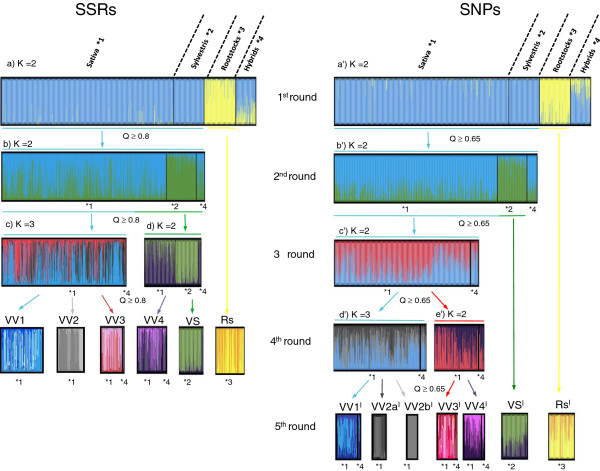
**Flow chart of hierarchical STRUCTURE analysis of the *Vitis *germplasm using 1085 unique accessions at 22 SSR loci and 1072 unique accessions at 353 SNP loci.** Plots generated with the DISTRUCT software based on the Q-matrix consensus permuted across 10 replications for each K using the CLUMPP software. In the first chart, samples of the four predefined groups are separated by black lines, while in subsequent charts, populations found by previous rounds of analysis are separated. Ultimately for the SSR and SNP data, respectively, there are: 1 cluster of rootstocks (Rs/Rs^I^), 1 cluster of *Vitis vinifera* sylvestris (VS/VS^I^) and 5 subclusters of cultivated grapevine: VV1, VV2, VV3, VV4/ VV1^I^, VV2^I^, VV3^I^, VV4^I^). Q – membership coefficient.

The population substructure within Sativa was best described through standard structure analysis at K = 5, where three possible subgroups were detected. However, additional subdivisions could not be excluded, since Wilcoxon test suggested K = 6 (four subgroups within Sativa) as the most plausible scenario when using SSRs (Figure 3). The genetic structure of cultivated grapevine has been influenced by human selection [14] and it can be largely understood as a complex pedigree, due to the vast number of higher order pedigree relationships [10]. Cryptic relatedness influences the study of the genetic structure, causing the overestimation of the probable subpopulations number (K) using standard methods [42]. A hierarchical approach was thus applied to delve deeper into the complex relationships of the germplasm. Initially, only individuals displaying a proportional membership > 0.8 in their primary population were considered in both marker data sets. However, this threshold was too stringent for the SNP data, due to the lower percentage assignment shown for K > 2 (data not shown). Since the percentage of individuals assigned to a subpopulation at K = 5 was similar for the SNP data with Q > 0.65 and for the SSR data with Q > 0.8 (Figure 4), the threshold of proportional membership for the hierarchical approach was set to > 0.65 for the SNP dataset. Using both datasets, Rootstocks grouped clearly in a distinct cluster (Rs) with a possible further subdivision into two subgroups (Figure 5).

Using the SSR set, a subsequent round (second round) of STRUCTURE separated most of the Sativa accessions from the group of Sylvestris. Following runs of STRUCTURE revealed a further stratification in the Sativa cluster into 3 groups (VV1, VV2 and VV3) and distinguished an additional group of Sativa accessions (VV4) from the Sylvestris genotypes (VS). Clusters VV1, VV2, VV3 and VV4 represent mainly Italian/Balkan wine grapes, Mediterranean table/wine grapes, Muscats (wine/table grapes) and Central European wine grapes, respectively. With the SNP dataset, the second round of STRUCTURE separated well the cultivated from the wild grapevines (VS^I^). At this point the threshold imposed on the SNP set excluded some Sativa accessions from further clustering (i.e. Pinot Noir, Gewürztraminer, Rhein Riesling, Aromriesling, Sauvignon, Sauvignonasse, Perle and Sacy among the true-to-type individuals). These accessions showed an admixed ancestry ≈ 0.5 of both Sativa and Sylvestris clusters and were grouped in VV4 when the SSR set was used. Following runs of STRUCTURE first separated the Sativa samples in two main groups and those were subsequently subdivided in five clusters (VV1^I^, VV2a^I^, VV2b^I^, VV3^I^, VV4^I^). Further clustering did not reveal anything new about the relationships amongst the accessions either because no individuals were strongly assigned (Q > 0.8 for SSRs and Q > 0.65 for SNPs) or a lack of background information on the samples did not allow the divisions to be independently supported. The clusters identified in the hierarchical and canonical STRUCTURE approach were in agreement, despite the different number of individuals assigned to the final populations (623 vs 831 for the SSR set and 443 vs 649 for the SNP set in the hierarchical and canonical STRUCTURE approach respectively). Moreover the hierarchical approach revealed the presence of an additional subgroup (VV2) not detected at K = 5. Thus, VS (VS^I)^, Rs and Rs^I^), VV3 (VV3^I^), and VV4 (VV4^I^) correspond to S4, S5, S2 and S3 respectively while VV1 (VV1^I^) and VV2 (VV2a^I^, VV2b^I^) were not separated at K = 5 and were grouped together in S1. Altogether, almost 97% of the individuals clustered in the hierarchical analysis were similarly clustered in the canonical approach at K = 5.

### Clusters composition and the origin of cultivated grapevine subpopulations

Clusters detected through the hierarchical approaches were consistent for both marker sets, despite the difference between the group VV2 (SSR) which was split into VV2a^I^ and VV2b^I^ when the SNP set was used. The considerable degree of uncertainty about the variety names of many samples in the collection limits the interpretation of this kind of result. Nevertheless, when only true-to-type accessions are considered it can be seen that SNPs made a distinction between Mediterranean grapevines of table grape cultivars related to ‘Sultanina’ (i.e. ‘Calmeria’, ‘Flame Seedless’) and Spanish wine grape cultivars (i.e. ‘Xarello’, ‘Macabeu’, ‘Parellada’, ‘Beba’). All the clusters identified using the SSR set contained more accessions compared to those defined based on the SNP set, with the greatest differences observed for groups VV1 (129) vs. VV1^I^ (51) and VV4 (112) vs. VV4^I^ (52) (Additional file 1: Table S7). This can be explained again by differences in allelic richness of these marker types, with lower assignment success for SNPs, even when the proportional membership threshold was reduced to 0.65. However, almost half of the accessions grouped in each Sativa cluster using the SNP set were also grouped accordingly when using the SSR set. Additionally, none of the unshared accessions could be found grouped in a different cluster, supporting the robustness of the clustering method.

Relatedness among samples influences the ability of STRUCTURE to correctly detect the genetic stratification of a germplasm [52,53]. This problem was partially overcome in the present study by investigating different criteria to find the best K in the “standard” STRUCTURE method, and then by applying a hierarchical approach. When using the former method most of the accessions sharing high order of pedigree relationships were not grouped into a specific cluster and were ultimately excluded. Thus, the final clusters contain mainly first degree pedigree relationships and represent the most plausible genetic structure of the germplasm investigated, being the smallest number of populations (K) “that captures the major structure of the data” [42]. STRUCTURE detected additional subpopulations within these groups as a consequence of sample relatedness. For instance, VV3 is further divided into offspring either of ‘Muscat of Alexandrie’ or ‘Moscato Bianco’ that are considered two of the oldest grape varieties still in existence. Moreover, this clustering substantially agrees with the classification of eco-geographic variation proposed by Negrul [54] and Levadoux [55] as well as with previous genetic structure analysis performed on a restricted number of cultivated (222) and wild (22) grapevines from a different germplasm [55]. According to Negrul [54], Italian and Greek wine grapes (VV1) belong to the group *pontica* and the French and German wine grapes (VV4) belong to the group *occidentalis*, whereas the Muscat table and wine cultivars (VV3) belong to the group *orientalis* (sub-proles *caspica*). The composition of the group VV2 is more heterogeneous since it includes table grape varieties related to ‘Sultanina’ (group *orientalis* sub-proles *antasiatica*) and some Spanish wine grapes, whose origin is still unknown. Aradhya et al. [14] reported similar results of grouping seedless type cultivars along with a number of southern European minor wine varieties into the “Mediterranean table-grape” cluster.

### Genetic diversity among clusters

**Figure 6:**
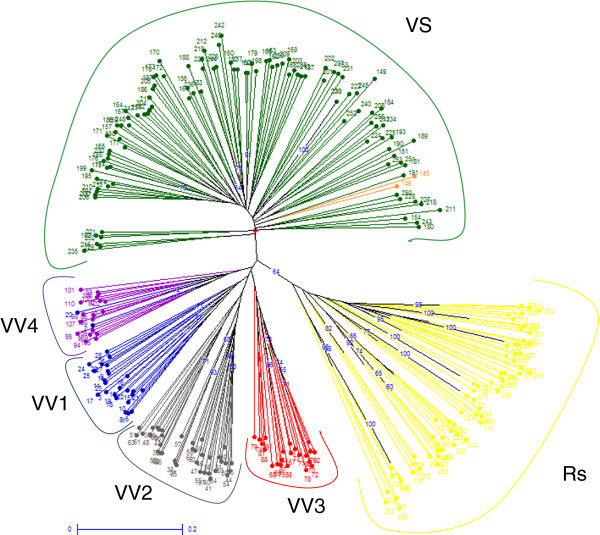
**Neighbour-joining dendrogram based on simple matching dissimilarity matrix calculated from the dataset of 22 SSRs across 330 genotypes clustered through the hierarchical STRUCTURE analysis.** Branch length is proportional to the distance between nodes. Bootstrap support ≥ 60 indicated along the branches represents the percent of times out of 10000 that two accessions grouped together during bootstrap analysis.

A neighbor joining unweighted tree was built based on SSR alleles, considering the 330 common accessions (109 Sativa, 110 Sylvestris and 111 Rootstocks) grouped in the final clusters by the hierarchical STRUCTURE approach with both marker sets (Figure 6). The dendrogram showed six distinct groups, supporting the consistency of the hierarchical clustering method.

Likewise, the pairwise F_ST_ analysis pointed out that these common clusters defined by hierarchical STRUCTURE represent statistically supported subpopulations (Additional file 1: Table S8). Very similar F_ST_ values were also found when considering all the accessions, grouped using the SNP and SSR marker sets separately (data not shown). As expected, the highest pairwise genetic differentiation was observed among clusters of Sylvestris and Rootstocks. In the case of the SNP set, the F_ST_ values are much higher compared to the SSR set when estimated for relationship with Rootstocks and Sylvestris. This could be due to bias introduced because the panel of SNPs was designed from a small sample of accessions, preventing detection of multiallele polymorphisms or because additional SNPs were located in the vicinity.

Based on SSRs, the group of Muscats (VV3) and the Mediterranean table and wine grapes (VV2) showed slightly higher F_ST_ values when compared to Sylvestris (F_ST_ = 0.18 and 0.17, respectively) than to accessions of Rootstocks (F_ST_ = 0.16 and 0.15, respectively). On the contrary the Italian-Greek wine grapes (VV1) and the French-German wine grapes (VV4) showed a lower level of differentiation (F_ST_ = 0.14 and 0.13, respectively) than the cluster of Sylvestris. This result might suggest a moderate genetic exchange between the wild grapevines and only a part of the cultivated grapevines, namely some of those belonging to the groups of *pontica* and *occidentalis*.

Despite a very low genetic differentiation (F_ST_ = 0.0048) among cultivars of Spanish and France-Central Europe origin being recently reported using SSRs [41], a moderate genetic differentiation (F_ST_ ranging from 0.09 to 0.15) among the cultivated grapevine clusters was detected based on SSRs in the present study. A moderate genetic differentiation was also found by using SNPs (F_ST_ ranging from 0.07 to 0.12) which is slightly higher compared to the results obtained among Western, Central and Eastern European cultivars (F_ST_ ranging from 0.02 to 0.051; [10]). Since the low genetic differentiation among cultivars reported up to now has been suggested to be a consequence of their complex pedigree [41], the higher values of F_ST_ estimated in the present study support the consistency of the intra-specific clusters detected by the hierarchical STRUCTURE approach.

### Genetic differentiation among clusters of grapevines

Archeological and historical evidences suggest that grape domestication took place in the Near East [56] and several studies have proposed the existence of secondary domestication events also along the Mediterranean basin [1,5,14,15]. The findings of Myles et al. [10] support an Eastern origin of most grape cultivars as well as the existence of introgression from wild germplasm in Western region. This was also suggested by de Andrès et al. [41] based on the genetic relationships between wild and cultivated Spanish grapevines and agrees with Negrul [54] and Levadoux [55] who suggest the cultivars of the *occidentalis* group possess wild morphological characters as evidence of spontaneous introgression from *V. sylvestris* into cultivated forms of the *pontica* group. Accordingly, our results put the *occidentalis* group (VV4), consisting of wine grapes mainly related to Pinot Noir and Traminer, closer to the wild samples compared to *pontica* (Italian and Greek) wine grapes. The origins of Traminer and Pinot Noir are poorly known and presumably ancient [57]. It has been suggested they could have arisen from hybridization between Roman grapes and local wild populations or from secondary domestication of the latter [58]. The ancient origin of Pinot Noir and Traminer may also be deduced from the evidence that many modern varieties are first-degree relatives of these cultivars [57,59].

The hierarchical STRUCTURE analysis grouped wild grapevines into a genetically distinct cluster, which, however, included some Sativa accessions (4 using the SSR set and 6 using the SNP set), while no additional subdivision of the cluster was detected. True-to-type Italian cultivars “Lambrusco a foglia frastagliata” (hermaphrodite) and “Lambrusco di Sorbara” (female) were common to both datasets. This apparently agrees with Levadoux et al. [55] who identified certain forms within the wild "Lambrusque" grapevines displaying traits commonly associated with domestication.

## Conclusions

In this study we provided standard marker profiles for the trueness-to-type assessment of grape cultivars and marker-assisted reduction of redundancy in a grape germplasm collection. Using 22 SSR and 384 SNP loci, we showed that both the marker systems are efficient for the evaluation of genetic diversity and population structure, and microsatellites turned out to be well suited for the construction of core collections. This is an important step to sustainable and effective use of available grape genetic resources in basic and applied research. For instance, the core collections may contribute to development of association mapping populations for investigating genotype-phenotype relationships. Our complementary approaches to the analysis of SSR and SNP datasets detected consistent inter- and intraspecific levels of germplasm stratification with four ancestral subpopulations of *V. vinifera* ssp. *sativa*. This is in accord with the eco-geographical origin of the cultivars.

## Methods

### Plant material and DNA extraction

A total of 2273 accessions of grape (*Vitis* spp.) were analyzed in this study. They belong to the FEM grape germplasm collection (ITA362), located in San Michele all'Adige, Italy (46°18’ N, 11°13’ E). All plants were grafted on the rootstock Kober 5BB in five replicates and trained according to the Guyot system. Each accession was classified to a certain category based on the collections record; thus the material consisted of 1659 cultivated grapevines, *V. vinifera* ssp. *sativa* (Sativa), 177 wild individuals of *V. vinifera* ssp. *sylvestris* (Sylvestris), 127 interspecific hybrids used for fruit production (Hybrids) and 310 accessions of rootstock varieties including wild non-*vinifera Vitis* species (Rootstocks). Based on phenological data recorded for every Sativa accession through visual inspection in the field, a set of 163 grapevine cultivars was previously sorted from the germplasm collection. This set can be considered representative with regard to the diverse timing of budburst, flowering, *véraison* and full ripening, and will be referred in this study as the “phenological core” (core P). The range in dates of the major growth stages observed in the core P since 2008 using the modified E-L system [60] is reported in Additional file 1: Table S2.

Detailed information about each accession is publicly available at the European *Vitis* Database [61].

Young leaf tissue of one field grown plant per accession was harvested and stored immediately in 96-well microtube plates. Two controls (Pinot Noir and Sangiovese cultivars) were added to each set of 94 accessions for DNA extraction and successive analyses. Total genomic DNA was isolated from freeze-dried tissue after grinding with the MM 300 Mixer Mill system (Retsch., Germany). DNA extraction was performed using the DNeasy 96 plant mini kit (QIAGEN, Germany). DNA was suspended in TE buffer (pH = 8) and digested with RNase A (QIAGEN) at 37°C for 1 h. Next the DNA samples were diluted to approximately 4 ng/μl before conducting PCR.

### SSR selection and genotyping

Twenty two SSR markers previously developed for grape, scattered over the genome, were chosen in this study (Additional file 1: Table S3): twenty SSR markers used by Laucou et al. [9] and the markers VrZAG62 and VrZAG79 [29]. This set includes the 9 SSR markers proposed by the European Project GrapeGen06 for the characterization of regional cultivars [11].

Nine multiplex panels of fluorescent-labeled microsatellite loci were used. Simultaneous PCR amplifications were carried out in a final volume of 12.5 μl containing 10 ng of genomic DNA, 0.25 mM of each dNTPs, 2 mM MgCl_2_, 1.5 U Taq DNA Polymerase (AmpliTaq, Gold™, Applied Biosystems, Foster City, CA). Depending on the locus, primer concentrations ranged from 0.2 to 0.6 μM. Reactions were performed on a GeneAmp PCR System 9700 using the following profile: a hot start of 95°C for 7 min, 30 amplification cycles of 45 sec at 95°C, 1 min at 54°C, 30 sec at 72°C, and a final extension step of 1 hour at 72°C. PCR products (0.5 μl) generated by two or three different fluorescence dye-labeled primers were mixed with 9.3 μl of formamide and 0.2 μl of the GeneScan™ 500 ROX® Size Standard (Applied Biosystems). The DNA fragments were denatured and size fractionated using capillary electrophoresis on an ABI 3130 Genetic Analyzer (Applied Biosystems). Subsequently, GeneMapper v3.5 (Applied Biosystems) was used for the allele size estimation. Rates of missing data (MD) were below 1% for markers included in the multiplex panels 1, 2, 3 and 4, while for the remaining loci MD were below 5% (for details see Additional file 1: Table S3).

### SNP selection and genotyping

The set of 384 SNPs used in this study was selected from informative data produced by previous SNP discovery and validation projects [19,39,62,63] and included 35 out of the 48 SNPs proposed by Cabezas et al. [12] for the identification of grapevine cultivars. The diverse discovery panels included 11 samples of ancient unrelated cultivars and wild *V. vinifera* for 164 SNPs found by Lijavetzky et al. [19], 10 cultivated *V. vinifera* and 7 wild *Vitis* species for 88 SNPs identified by Myles et al. [63] and 10 complex interspecific grape hybrids for 9 SNPs discovered by Zyprian et al. (in preparation). Of the remaining SNPs, 122 were identified in the cultivar Pinot Noir [62] and one was discovered in the Muscat family of grapevine [39]. All these SNPs meet the criteria of having enough upstream and downstream sequence information and of absence of other known SNPs in their vicinity. A designability score calculated for each SNP by Illumina was higher than 0.6, and this predicted high assay conversion rates. Genetic map positions were known for 257 of these SNPs, with 20, 9, 11, 17, 10, 14, 18, 23, 12, 16, 8, 11, 12, 15, 10, 10, 7, 20 and 14 loci placed on linkage groups 1 to 19, respectively. Genomic locations of SNPs on the reference grape genome (inbred Pinot Noir, 8X) are shown in Additional file 6. Chromosomal location and allelic variants for each SNP marker are reported in Additional file 1: Table S4. The genotyping was performed on an Illumina BeadStation 500 G system at Parco Tecnologico Padano (Lodi, Italy), using the protocol supported by Illumina.

### Genetic diversity within and among groups of the germplasm collection

The genotypic data were subjected to various within and among groups genetic diversity measures, such as mean number of alleles per locus (a), number of effective alleles (A_E_, the number of equally frequent alleles required to give the observed level of heterozygosity; [64]), levels of observed (H_O_) and expected (H_E_) heterozygosity [65], genetic differentiation (F_ST_) and the fixation index (F, inbreeding coefficient; [66]). All these calculations, together with values of PI (the probability that two individuals in the population share the same genotype, [67]) and MAF (minor allele frequency), were performed using GenAlex 6.41 [68] and GENETIX [69].

### Construction of genetic core collections

Core collections are subsamples of larger germplasm collections and include accessions chosen to represent the majority of the genetic diversity contained in these larger collections [70]. To construct the genetic core collections we used the M (maximization) method, suggested by Schoen and Brown [71], implemented in the MSTRAT software [72]. The M strategy selects specific combinations of accessions while maximizing the number of observed alleles at each marker locus and the MSTRAT uses iterative procedures to select samples with the highest allelic diversity. The final number of iterations per MSTRAT run was 150, while the number of repetitions for core sampling was 100. Putative core collections exhibiting the same allelic richness were ranked using Nei’s diversity index [73]. The accessions that were most often present in the 100 replicates were retained as the final core collection. The efficiency of the strategy was assessed by comparing the total number of alleles captured using MSTRAT in samples of increasing size to the number of alleles captured in randomly chosen collections of equal size (ten independent samplings).

### Analysis of population structure

The genetic structure of the germplasm collection was analyzed performing Principal Coordinate Analysis (PCoA) and Principal Component Analysis (PCA) implemented in the programs GenAlex 6.41 [68] and GenoDive 2.0b21 [74], and by using STRUCTURE 2.1 software [47,52]. PCoA was based on standardized covariance of genetic distances calculated for codominant markers while PCA was calculated by using the variance-covariance matrix of allele frequencies. Missing data were replaced by alleles randomly picked from the allele pool of each population. To avoid bias in the analysis of population structure, pairwise linkage disequilibrium (LD) between SNPs was evaluated using the software TASSEL v2.1 [75] by setting 1000 permutations. The model-based approach implemented in STRUCTURE 2.1 was used at the Bioportal server [76]. This software applies a Bayesian clustering algorithm to identify subpopulations, assign individuals to them, and estimate the population allele frequencies. STRUCTURE sorts individuals into K clusters, according to their genetic similarity. The best K is chosen based on the estimated membership coefficients (Q) for each individual in each cluster. Ten independent runs for K values ranging from 1 to 20 were performed with a burn-in length of 1000000 followed by 1500000 iterations. The admixture model was applied and no prior population information was used. The log-probability of the data, given for each value of K, was calculated and compared across the range of K. The software CLUMPP 1.1 [77] was used to find optimal alignments of independent runs and the output was used directly as input into a program for cluster visualization DISTRUCT 1.1 [78]. The optimal subpopulation model was investigated in several ways: (1) by applying the informal pointers (i.e. the plateau criterion) proposed by Pritchard et al. [42], (2) by evaluating L(K), the log probability values from ten runs at each K, using non-parametric Wilcoxon test, as implemented in the R software package Rcmdr [79], (3) by considering 3ΔK, a second order rate change with respect to K, as defined in Evanno et al. [44], and (4) by plotting the log probability L(K) and ΔK of the data over ten runs, as implemented in STRUCTURE HARVESTER [80]. In addition the following parameters were calculated using the R-script Structure-Sum [81]: the average similarity coefficients for different simulations within each preset K [45] and the extent of membership in a single cluster measured by the clusteredness statistic [82]. Furthermore, a “hierarchical STRUCTURE analysis” [83] was applied in this study by running STRUCTURE subsequently on partitioned data, as suggested by Pritchard et al. [42], using only the individuals suspected of being subdivided into different clusters. For this approach the ΔK method [44] was used in adjudication for the best K and the individuals with a proportional membership Q > 0.8 (SSRs) and Q > 0.65 (SNPs) in their primary population were considered in the subsequent analysis.

The consistency of the clusters identified through the hierarchical STRUCTURE approach was tested by pairwise F_ST_ analysis [84]. In addition, an unweighted neighbour-joining tree was constructed based on dissimilarities between 330 accessions (calculated from 22 SSRs), and ten thousand bootstrap replicates were performed using the Darwin software package v5.0148 [85].

## Competing interests

The authors declare that they have no competing interests.

## Authors’ contributions

FE participated in the design of the study, constructed core collections, performed population structure analysis, and drafted part of the manuscript. SL carried out SSR analysis and phenotyping, and managed the data produced during the project. LG carried out genetic diversity analyses and drafted part of the manuscript. VC provided lab assistance and managed the SNP dataset. MS provided germplasm records and checked accession plants for inconsistencies. MT, SM, JMMZ and EZ participated in the SNP selection and helped in the discussion of results. FMM participated in the design of the study and produced the non-redundant SSR dataset. MSG conceived the study, participated in its design and coordination, and wrote part of the manuscript. All authors read and approved the final manuscript.

## Supplementary Material

Additional file 1: Table S1 Groups of accessions with the identical SSR profile that included true-to-type Italian varieties. Names of accessions registered as synonymous to the true-to-type prime name are indicated in bold (*Vitis* International Variety Catalogue http://www.vivc.de). **Table S2.** Minimum and maximum dates of the beginning of phenological stages for the “phenological core collection” in the three growing seasons (2008-2010). The E-L numbers indicate major vine growth stages according to the modified Eichhorn-Lorenz system [60]. **Table S3.** SSR markers and multiplex PCR conditions, allele size range and marker profiles of the grapevine cultivars (Pinot noir and Sangiovese) used as internal control for genotyping. ^a^ SSR markers with the same number were amplified in a single PCR mix (all primers pooled in the PCR mix). * Reference set of SSR markers used for cultivar identification. **Table S4.** A total of 384 SNPs selected for genotyping of the FEM grape germplasm collection. Chr - chromosome carrying the SNP according to the reference grapevine genome (Pinot Noir, 8x); LG – linkage group; MAF – minor allele frequency. Source of markers: No. 1-122: [62]; No. 123-286: [19]; No. 287-374: [63]; 375-383: Zyprian et al. (in preparation); 384: [39]. **Table S5.** Summary statistics of genetic variation at each of the 22 SSR loci in the FEM grape germplasm collection. Total – pooled sample treated as a single population; N – sample size; n – mean sample size over loci; A – number of different alleles; a – mean number of alleles per locus; A_E_ – effective number of alleles; A_pr_ – number of alleles unique to a single population; H_O_ – observed heterozygosity; H_E_ – unbiased expected heterozygosity; F – fixation index (inbreeding coefficient). **Table S6.** Percent population assignment based on SSR and SNP marker datasets. Each value gives the percentage of individuals that had ≥0.8 membership in a subpopulation using the STRUCTURE analysis (K=2 to 6, with SSR or SNP dataset). **Table S7.** Groups of *V. vinifera* ssp. *sativa* inferred by hierarchical STRUCTURE using SSR and SNP datasets. Listed are the accessions common in the four clusters distinguished using SSR and SNP datasets (i.e. in VV1 and VV1^I^, VV2 and VV2^I^, VV3 and VV3^I^, VV4 and VV4^I^). The true-to-type samples from these four groups are indicated in bold. **Table S8.** Common cluster pairwise F_ST_ estimates (P=0.00, 1000 permutations). Click here for file

Additional file 2 **Redundancy curves developed for genetic core collections G-58 and G-110 using the M-method (in blue) and random sampling (in red) with standard deviations, captured in ten independent sampling runs.** Plot shows the accumulation of allelic diversity with increasing core size. The core G-110 obtained using the M-method was built considering samples from the core G-58 as fixed. Click here for file

Additional file 3 **Estimated number of clusters obtained with STRUCTURE for K values from 1 to 20 using SSR data.** Graphical representation of (a) estimated mean L(K) and (b) its derivative statistics ΔK. (c) Table summarizing parameters of different STRUCTURE simulations performed for each preset K: mean likelihoods of models, mean similarity coefficients, clusteredness, and their standard deviations, ΔK and significance of Wilcoxon test. Click here for file

Additional file 4 **Estimated number of clusters obtained with STRUCTURE for K values from 1 to 20 using SNP data.** Graphical representation of (a) estimated mean L(K) and (b) its derivative statistics ΔK. (c) Table summarizing parameters of different STRUCTURE simulations performed for each preset K: mean likelihoods of models, mean similarity coefficients, clusteredness, and their standard deviations, ΔK and significance of Wilcoxon test. Click here for file

Additional file 5 **Neighbour-joining tree and inferred population structure of the grape germplasm collection, calculated from the dataset of 22 SSR markers and 353 SNPs across 1146 individuals using structure analysis (K=5).** Each individual is represented by a line partitioned in five coloured segments (the individual’s estimated membership fractions to each one of the five clusters). Threshold of the membership coefficient Q was 0.80 for the SSR dataset and 0.65 for the SNP dataset. Click here for file

Additional file 6 **Physical position of SNP and SSR markers.** The map shows the position (in megabases) of SNPs (in black) and SSRs (in red) for each chromosome within the 8X reference genome. Markers with unknown or uncertain physical position are not shown. Click here for file
