# Supplementary figures and images for: Genetic diversity and population structure assessed by SSR and SNP markers in a large germplasm collection of grape
Source: BMC Plant Biol. 2013 Mar 7;13:39. doi: 10.1186/1471-2229-13-39 (PMC3610244; doi:10.1186/1471-2229-13-39)

## Slide 1
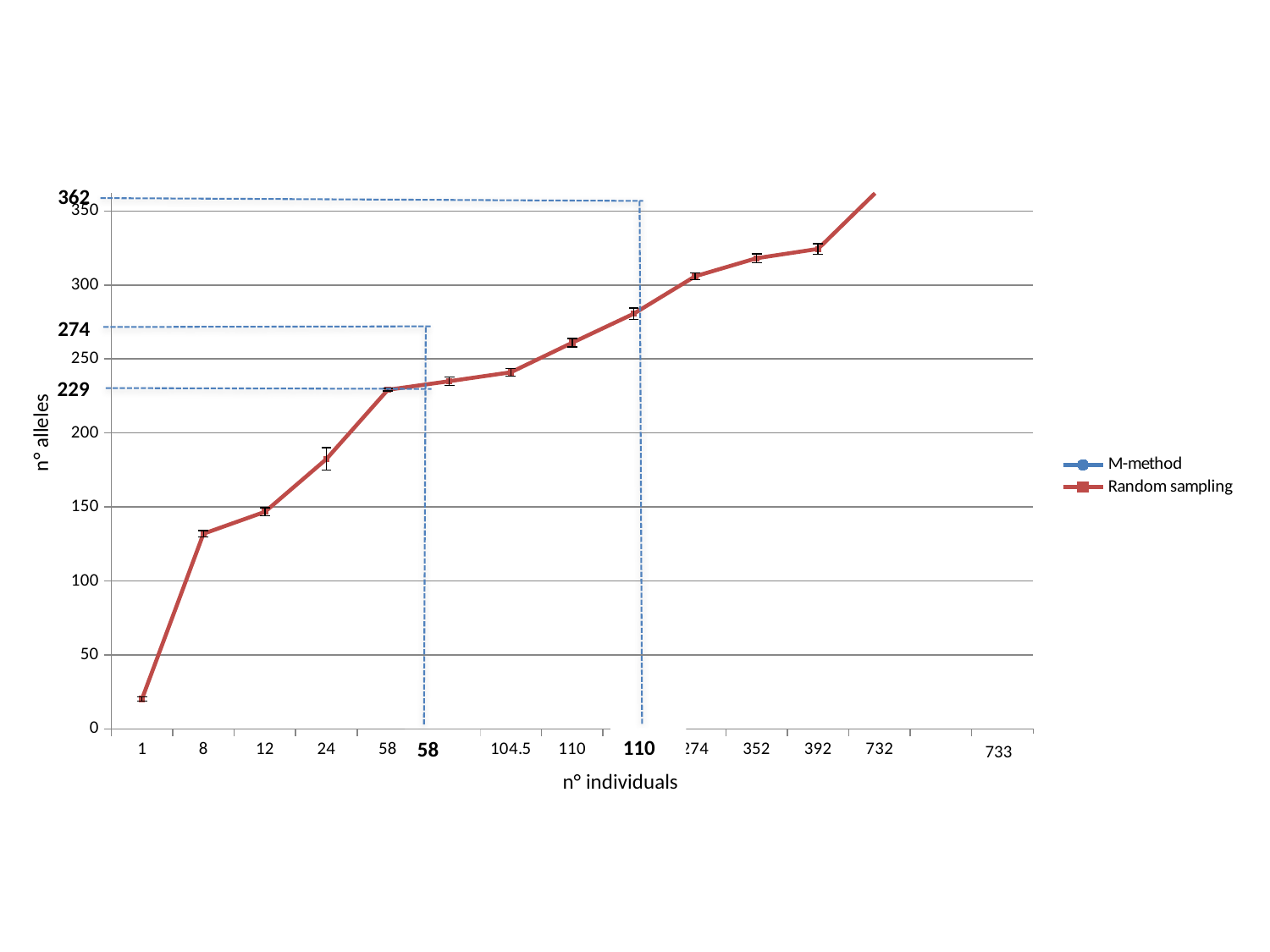

362
### Chart
| Category | | |
|---|---|---|
| 1 | 20.9 | 20.2 |
| 8 | 193.9 | 131.9 |
| 12 | 224.0 | 146.7 |
| 24 | 259.1 | 182.4 |
| 58 | 274.0 | 229.2 |
| 78.5 | 332.8 | 235.0 |
| 104.5 | 338.4 | 241.0 |
| 110 | 362.0 | 261.0 |
| 196 | 362.0 | 280.6 |
| 274 | 362.0 | 305.9 |
| 352 | 362.0 | 318.1 |
| 392 | 362.0 | 324.4 |
| 732 | 362.0 | 365.0 |274
229
n° alleles
110
58
733
n° individuals

Supplement: Additional file 2 — Redundancy curves developed for genetic core collections G-58 and G-110 using the M-method (in blue) and random sampling (in red) with standard deviations, captured in ten independent sampling runs. Plot shows the accumulation of allelic diversity with increasing core size. The core G-110 obtained using the M-method was built considering samples from the core G-58 as fixed. [file 1471-2229-13-39-S2.pptx]

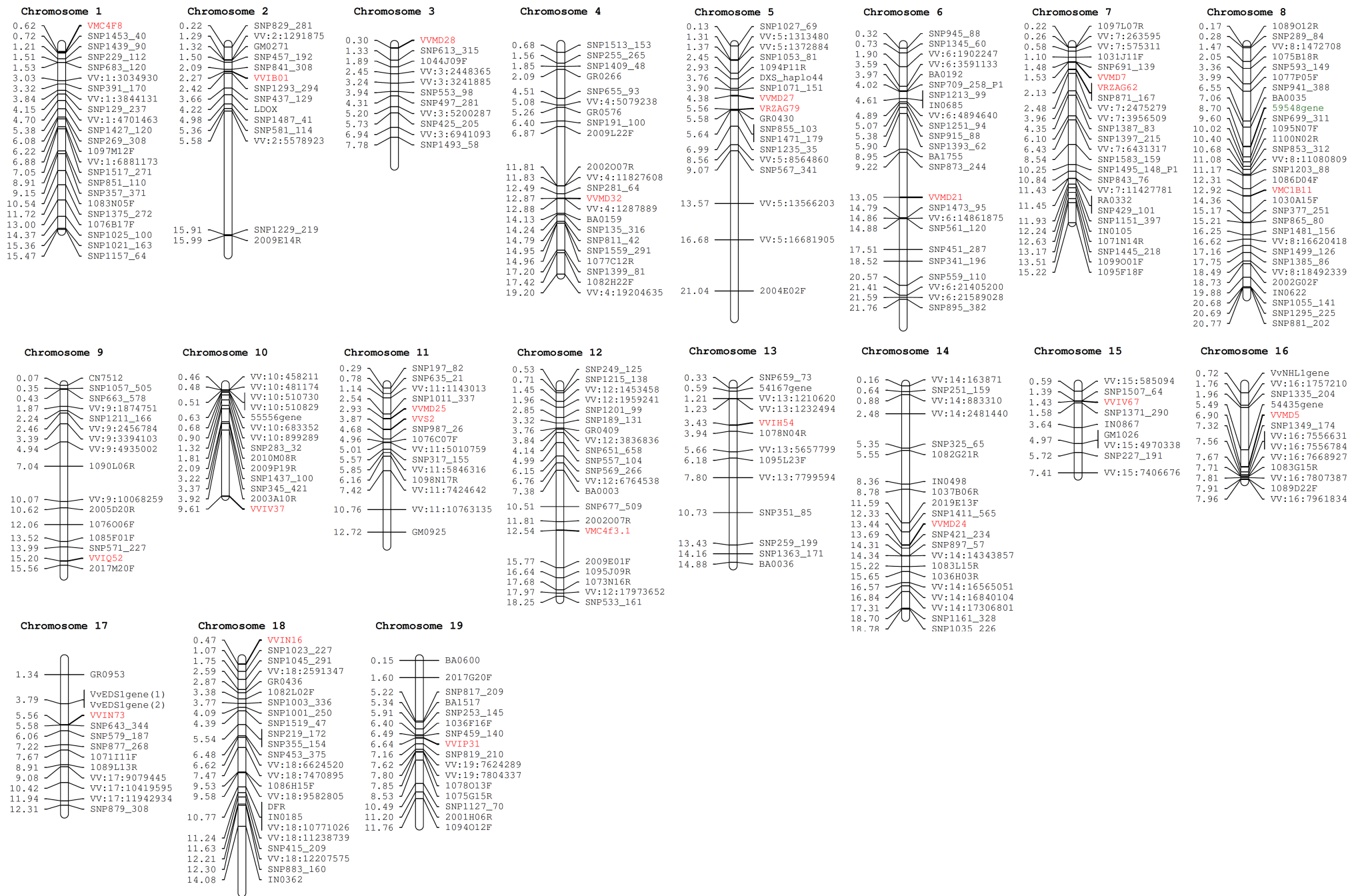

Supplement: Additional file 6 — Physical position of SNP and SSR markers. The map shows the position (in megabases) of SNPs (in black) and SSRs (in red) for each chromosome within the 8X reference genome. Markers with unknown or uncertain physical position are not shown. [file 1471-2229-13-39-S6.pdf]
