# Supplementary material for: Genetic diversity and population structure assessed by SSR and SNP markers in a large germplasm collection of grape
Source: BMC Plant Biol. 2013 Mar 7;13:39. doi: 10.1186/1471-2229-13-39 (PMC3610244; doi:10.1186/1471-2229-13-39)
Supplement: Additional file 3 — Estimated number of clusters obtained with STRUCTURE for K values from 1 to 20 using SSR data. Graphical representation of (a) estimated mean L(K) and (b) its derivative statistics ΔK. (c) Table summarizing parameters of different STRUCTURE simulations performed for each preset K: mean likelihoods of models, mean similarity coefficients, clusteredness, and their standard deviations, ΔK and significance of Wilcoxon test. [file 1471-2229-13-39-S3.pptx]

## Slide 1
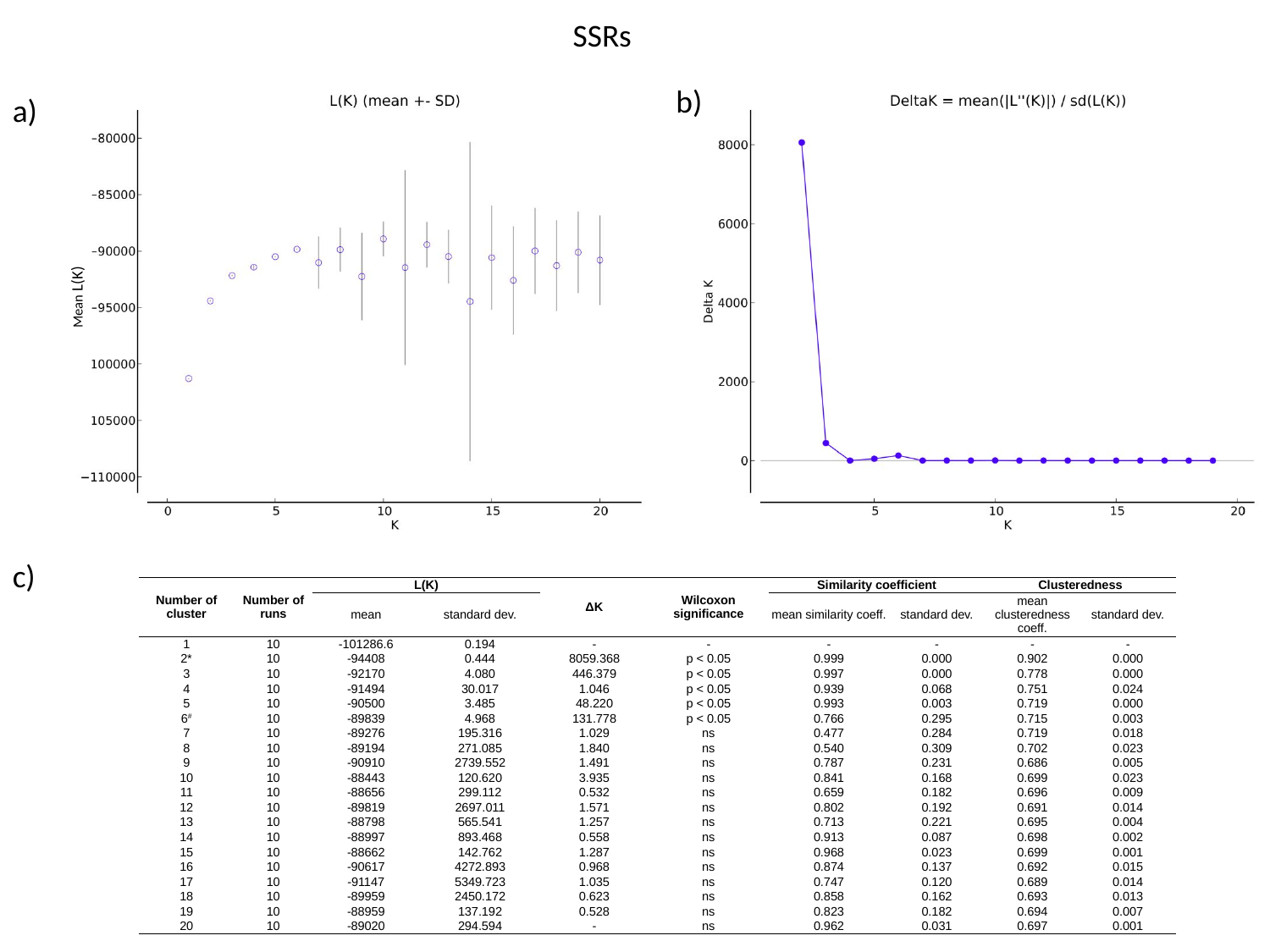

SSRs
b)
a)
Mean L(K)
c)
| Number of cluster | Number of runs | L(K) | | ΔK | Wilcoxon significance | Similarity coefficient | | Clusteredness | |
| --- | --- | --- | --- | --- | --- | --- | --- | --- | --- |
| | | mean | standard dev. | | | mean similarity coeff. | standard dev. | mean clusteredness coeff. | standard dev. |
| 1 | 10 | -101286.6 | 0.194 | - | - | - | - | - | - |
| 2\* | 10 | -94408 | 0.444 | 8059.368 | p < 0.05 | 0.999 | 0.000 | 0.902 | 0.000 |
| 3 | 10 | -92170 | 4.080 | 446.379 | p < 0.05 | 0.997 | 0.000 | 0.778 | 0.000 |
| 4 | 10 | -91494 | 30.017 | 1.046 | p < 0.05 | 0.939 | 0.068 | 0.751 | 0.024 |
| 5 | 10 | -90500 | 3.485 | 48.220 | p < 0.05 | 0.993 | 0.003 | 0.719 | 0.000 |
| 6# | 10 | -89839 | 4.968 | 131.778 | p < 0.05 | 0.766 | 0.295 | 0.715 | 0.003 |
| 7 | 10 | -89276 | 195.316 | 1.029 | ns | 0.477 | 0.284 | 0.719 | 0.018 |
| 8 | 10 | -89194 | 271.085 | 1.840 | ns | 0.540 | 0.309 | 0.702 | 0.023 |
| 9 | 10 | -90910 | 2739.552 | 1.491 | ns | 0.787 | 0.231 | 0.686 | 0.005 |
| 10 | 10 | -88443 | 120.620 | 3.935 | ns | 0.841 | 0.168 | 0.699 | 0.023 |
| 11 | 10 | -88656 | 299.112 | 0.532 | ns | 0.659 | 0.182 | 0.696 | 0.009 |
| 12 | 10 | -89819 | 2697.011 | 1.571 | ns | 0.802 | 0.192 | 0.691 | 0.014 |
| 13 | 10 | -88798 | 565.541 | 1.257 | ns | 0.713 | 0.221 | 0.695 | 0.004 |
| 14 | 10 | -88997 | 893.468 | 0.558 | ns | 0.913 | 0.087 | 0.698 | 0.002 |
| 15 | 10 | -88662 | 142.762 | 1.287 | ns | 0.968 | 0.023 | 0.699 | 0.001 |
| 16 | 10 | -90617 | 4272.893 | 0.968 | ns | 0.874 | 0.137 | 0.692 | 0.015 |
| 17 | 10 | -91147 | 5349.723 | 1.035 | ns | 0.747 | 0.120 | 0.689 | 0.014 |
| 18 | 10 | -89959 | 2450.172 | 0.623 | ns | 0.858 | 0.162 | 0.693 | 0.013 |
| 19 | 10 | -88959 | 137.192 | 0.528 | ns | 0.823 | 0.182 | 0.694 | 0.007 |
| 20 | 10 | -89020 | 294.594 | - | ns | 0.962 | 0.031 | 0.697 | 0.001 |
