# Supplementary material for: Genetic diversity and population structure assessed by SSR and SNP markers in a large germplasm collection of grape
Source: BMC Plant Biol. 2013 Mar 7;13:39. doi: 10.1186/1471-2229-13-39 (PMC3610244; doi:10.1186/1471-2229-13-39)
Supplement: Additional file 4 — Estimated number of clusters obtained with STRUCTURE for K values from 1 to 20 using SNP data. Graphical representation of (a) estimated mean L(K) and (b) its derivative statistics ΔK. (c) Table summarizing parameters of different STRUCTURE simulations performed for each preset K: mean likelihoods of models, mean similarity coefficients, clusteredness, and their standard deviations, ΔK and significance of Wilcoxon test. [file 1471-2229-13-39-S4.pptx]

## Slide 1
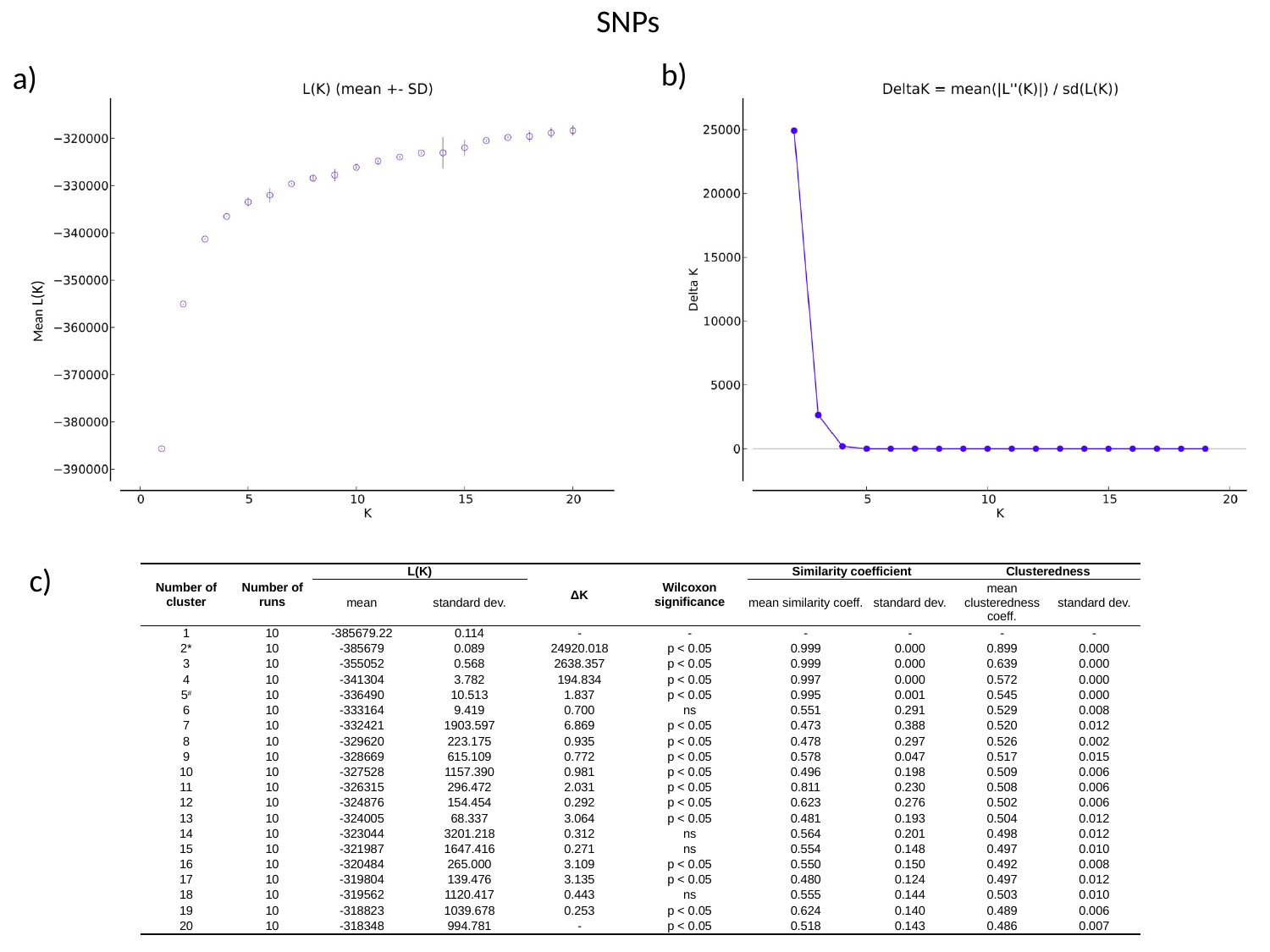

SNPs
b)
a)
Mean L(K)
c)
| Number of cluster | Number of runs | L(K) | | ΔK | Wilcoxon significance | Similarity coefficient | | Clusteredness | |
| --- | --- | --- | --- | --- | --- | --- | --- | --- | --- |
| | | mean | standard dev. | | | mean similarity coeff. | standard dev. | mean clusteredness coeff. | standard dev. |
| 1 | 10 | -385679.22 | 0.114 | - | - | - | - | - | - |
| 2\* | 10 | -385679 | 0.089 | 24920.018 | p < 0.05 | 0.999 | 0.000 | 0.899 | 0.000 |
| 3 | 10 | -355052 | 0.568 | 2638.357 | p < 0.05 | 0.999 | 0.000 | 0.639 | 0.000 |
| 4 | 10 | -341304 | 3.782 | 194.834 | p < 0.05 | 0.997 | 0.000 | 0.572 | 0.000 |
| 5# | 10 | -336490 | 10.513 | 1.837 | p < 0.05 | 0.995 | 0.001 | 0.545 | 0.000 |
| 6 | 10 | -333164 | 9.419 | 0.700 | ns | 0.551 | 0.291 | 0.529 | 0.008 |
| 7 | 10 | -332421 | 1903.597 | 6.869 | p < 0.05 | 0.473 | 0.388 | 0.520 | 0.012 |
| 8 | 10 | -329620 | 223.175 | 0.935 | p < 0.05 | 0.478 | 0.297 | 0.526 | 0.002 |
| 9 | 10 | -328669 | 615.109 | 0.772 | p < 0.05 | 0.578 | 0.047 | 0.517 | 0.015 |
| 10 | 10 | -327528 | 1157.390 | 0.981 | p < 0.05 | 0.496 | 0.198 | 0.509 | 0.006 |
| 11 | 10 | -326315 | 296.472 | 2.031 | p < 0.05 | 0.811 | 0.230 | 0.508 | 0.006 |
| 12 | 10 | -324876 | 154.454 | 0.292 | p < 0.05 | 0.623 | 0.276 | 0.502 | 0.006 |
| 13 | 10 | -324005 | 68.337 | 3.064 | p < 0.05 | 0.481 | 0.193 | 0.504 | 0.012 |
| 14 | 10 | -323044 | 3201.218 | 0.312 | ns | 0.564 | 0.201 | 0.498 | 0.012 |
| 15 | 10 | -321987 | 1647.416 | 0.271 | ns | 0.554 | 0.148 | 0.497 | 0.010 |
| 16 | 10 | -320484 | 265.000 | 3.109 | p < 0.05 | 0.550 | 0.150 | 0.492 | 0.008 |
| 17 | 10 | -319804 | 139.476 | 3.135 | p < 0.05 | 0.480 | 0.124 | 0.497 | 0.012 |
| 18 | 10 | -319562 | 1120.417 | 0.443 | ns | 0.555 | 0.144 | 0.503 | 0.010 |
| 19 | 10 | -318823 | 1039.678 | 0.253 | p < 0.05 | 0.624 | 0.140 | 0.489 | 0.006 |
| 20 | 10 | -318348 | 994.781 | - | p < 0.05 | 0.518 | 0.143 | 0.486 | 0.007 |
