# Supplementary material for: Genetic diversity and population structure assessed by SSR and SNP markers in a large germplasm collection of grape
Source: BMC Plant Biol. 2013 Mar 7;13:39. doi: 10.1186/1471-2229-13-39 (PMC3610244; doi:10.1186/1471-2229-13-39)
Supplement: Additional file 5 — Neighbour-joining tree and inferred population structure of the grape germplasm collection, calculated from the dataset of 22 SSR markers and 353 SNPs across 1146 individuals using structure analysis (K=5). Each individual is represented by a line partitioned in five coloured segments (the individual’s estimated membership fractions to each one of the five clusters). Threshold of the membership coefficient Q was 0.80 for the SSR dataset and 0.65 for the SNP dataset. [file 1471-2229-13-39-S5.pptx]

## Slide 1
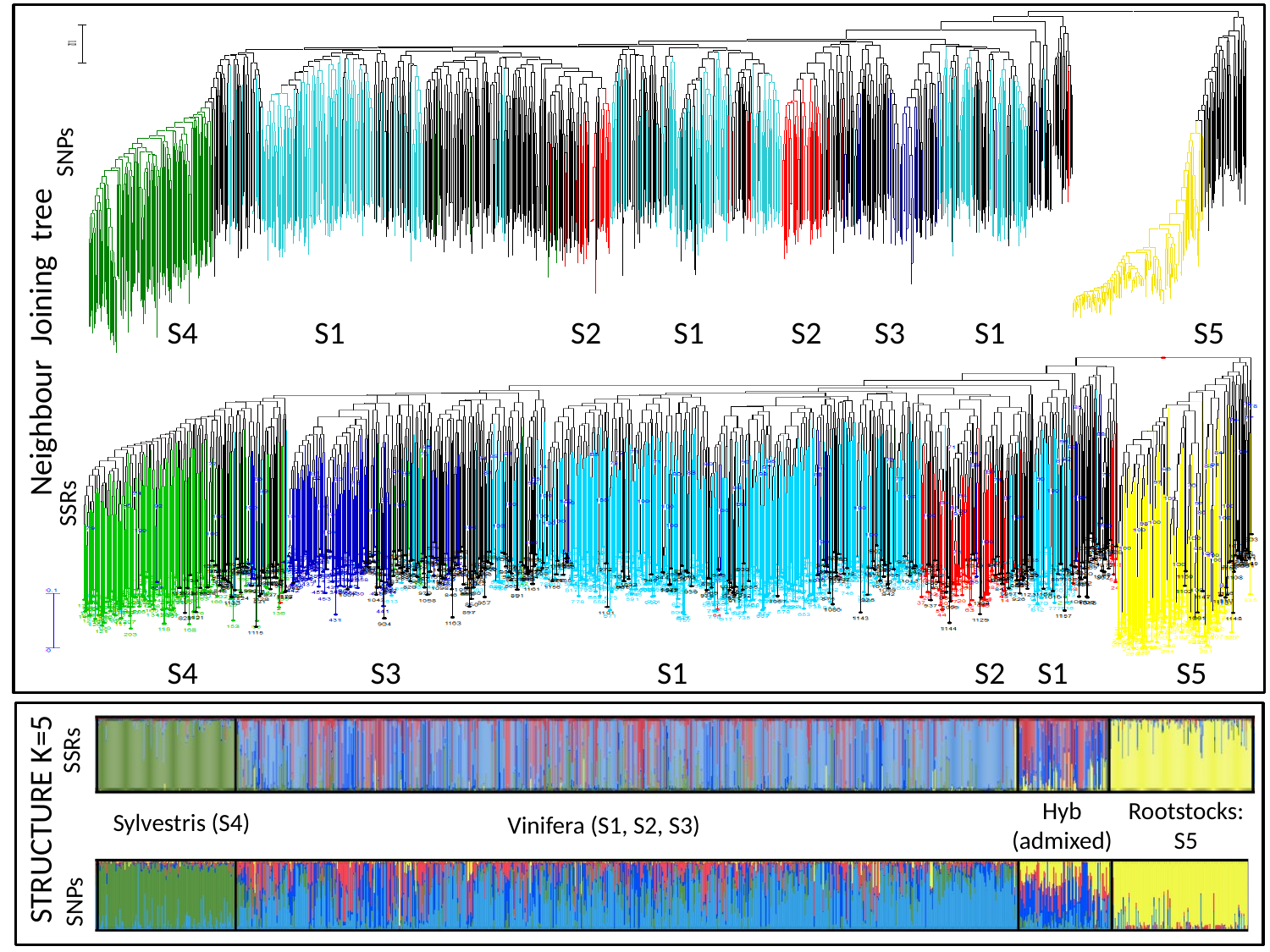

SNPs
Neighbour Joining tree
S1
S2
S1
S4
S3
S2
S1
S5
SSRs
S4
S1
S2
S3
S1
S5
SSRs
STRUCTURE K=5
Hyb (admixed)
Rootstocks: S5
 Sylvestris (S4)
Vinifera (S1, S2, S3)
SNPs
